# Individualized antibiotic dosage regimens for patients with augmented renal clearance

**DOI:** 10.3389/fphar.2023.1137975

**Published:** 2023-07-26

**Authors:** A-Xi Shi, Qiang Qu, Hai-Hui Zhuang, Xin-Qi Teng, Wei-Xin Xu, Yi-Ping Liu, Yi-Wen Xiao, Jian Qu

**Affiliations:** ^1^ Department of Pharmacy, The Second Xiangya Hospital, Institute of Clinical Pharmacy, Central South University, Changsha, China; ^2^ Department of Pharmacy, The First Hospital of Lanzhou University, Lanzhou, China; ^3^ Department of Pharmacy, Xiangya Hospital, Central South University, Changsha, China; ^4^ National Clinical Research Center for Geriatric Disorders, Xiangya Hospital, Central South University, Changsha, China; ^5^ Hunan Key Laboratory of the Research and Development of Novel Pharmaceutical Preparations, Changsha Medical University, Changsha, China

**Keywords:** augmented renal clearance, antibiotic, pharmacokinetics, pharmacodynamics, individualized

## Abstract

**Objectives:** Augmented renal clearance (ARC) is a state of enhanced renal function commonly observed in 30%–65% of critically ill patients despite normal serum creatinine levels. Using unadjusted standard dosing regimens of renally eliminated drugs in ARC patients often leads to subtherapeutic concentrations, poor clinical outcomes, and the emergence of multidrug-resistant bacteria. We summarized pharmaceutical, pharmacokinetic, and pharmacodynamic research on the definition, underlying mechanisms, and risk factors of ARC to guide individualized dosing of antibiotics and various strategies for optimizing outcomes.

**Methods:** We searched for articles between 2010 and 2022 in the MEDLINE database about ARC patients and antibiotics and further provided individualized antibiotic dosage regimens for patients with ARC.

**Results:** 25 antibiotic dosage regimens for patients with ARC and various strategies for optimization of outcomes, such as extended infusion time, continuous infusion, increased dosage, and combination regimens, were summarized according to previous research.

**Conclusion:** ARC patients, especially critically ill patients, need to make individualized adjustments to antibiotics, including dose, frequency, and method of administration. Further comprehensive research is required to determine ARC staging, expand the range of recommended antibiotics, and establish individualized dosing guidelines for ARC patients.

## 1 Introduction

The concept of augmented renal clearance (ARC) was proposed by Udy et al. (Udy et al., 2010) in 2010, who defined a phenomenon in critically ill patients characterized by enhanced creatinine clearance (CrCl) and removal of renally eliminated circulating solutes (Udy et al., 2010; Udy et al., 2011). Using conventional dosing regimens for ARC patients may lead to suboptimal drug exposure and therapeutic failure (Udy et al., 2010; Udy et al., 2011; Baptista et al., 2019; Luo et al., 2021). This review discusses the characteristics of ARC, its influence on antibiotic therapy, and dose optimization strategies to improve outcomes.

## 2 Definition of ARC

Augmented renal clearance (ARC) is clinically defined as creatinine clearance (CrCl) of more than 130 mL/min/1.73m2 (Udy et al., 2011; Mahmoud and Shen, 2017). Other threshold values of 120, 150, and 160 mL/min/1.73m2 have additionally been suggested (Udy et al., 2010; Campassi et al., 2014; Carrie et al., 2019). CrCl is measured through urine collection followed by evaluation of the creatinine concentration using the formula: creatinine clearance = [urine creatinine concentration × urine volume]/[serum creatinine concentration × time], abbreviated as CrCl = [UCr×Uvol]/[SCr×Tmin](Cherry et al., 2002; Claus et al., 2013; Baptista et al., 2014). Measurements of CrCl at 8, 12, or 24 h are more accurate in critically ill patients with normal serum creatinine (sCr) levels since glomerular filtration rate (GFR) values obtained using Cockroft-Gault (CG), Chronic Kidney Disease Epidemiology Collaboration (CKD-EPI) and Modification of Diet in Renal Disease Study (MDRD) formulae underestimate renal function in ARC (Baptista et al., 2014; Morbitzer et al., 2019). Several scoring systems have additionally been developed to predict ARC (Udy et al., 2013; Barletta et al., 2017; Gijsen et al., 2020; Saito et al., 2020). However, validation of the accuracy of the models and unification of standards for definitive diagnosis of ARC remains an urgent clinical need.

## 3 Mechanism of ARC

**FIGURE 1:**
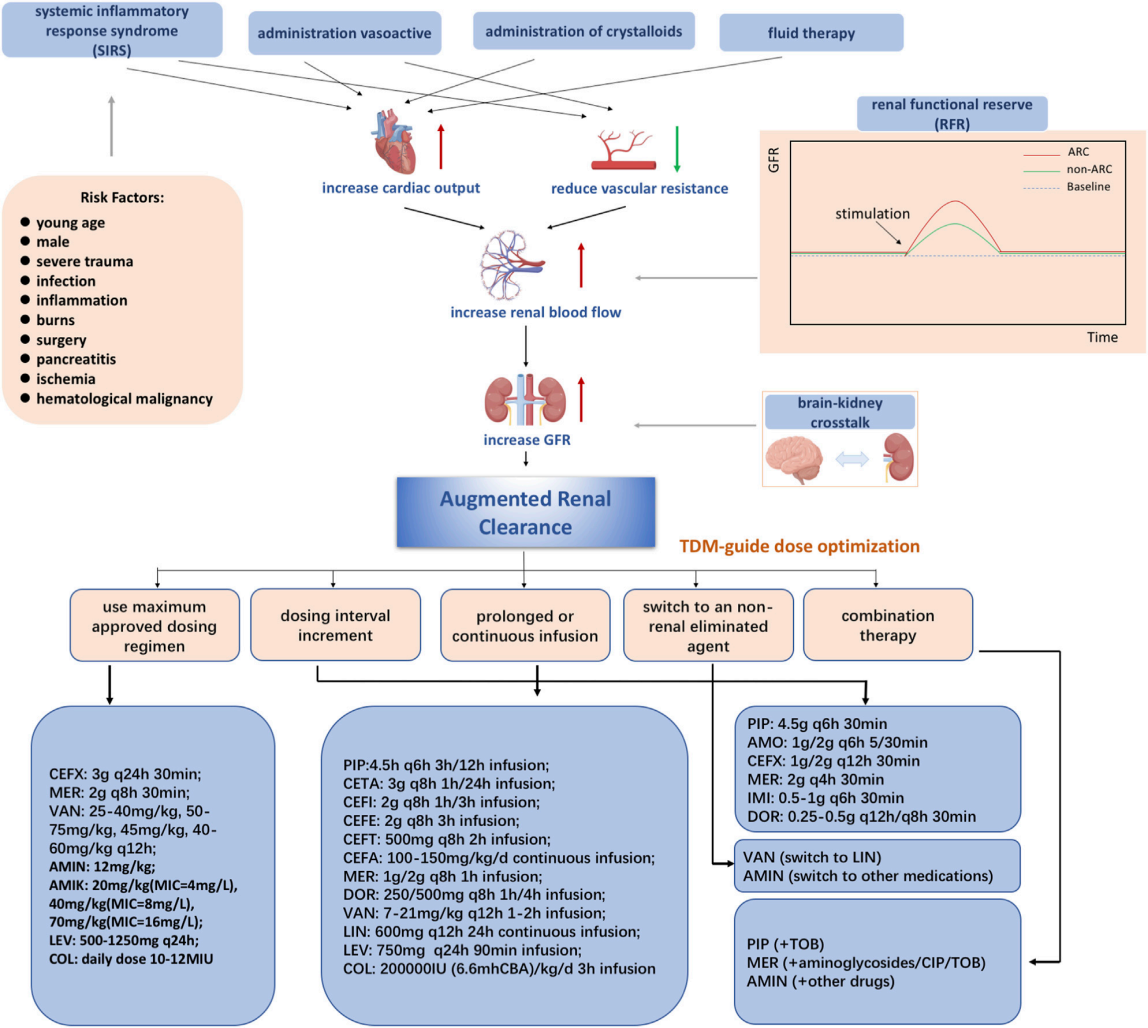
Mechanisms of augmented renal clearance Abbreviations: CEFX, ceftriaxone; MER, meropenem; VAN, vancomycin; AMIN, aminoglycoside; AMIK, amikacin; LEV, levofloxacin; COL, colistin; PIP, piperacillin-tazobactam; AMO, amoxicillin/clavulanic acid; IMI, imipenem; DOR, doripenem; CETA, ceftolozane/tazobactam; CEFI, cefiderocol; CEFE, cefepime; CEFT, ceftobiprole; CIP, ciprofloxacin; CEFA, cefazolin; LIN, linezolid; TOB, tobramycin.

The mechanism of ARC in patients has not been clearly defined and needs to be studied more. Generally, the underlying mechanisms of ARC are characterized by a hyperkinetic state, elevated cardiac output, and greater blood flow to major organs, which may lead to increased kidney perfusion (Bilbao-Meseguer et al., 2018). Three main possible mechanisms are proposed based on the comprehensive analysis of previous studies (shown in Figure 1). One possible mechanism is systemic inflammatory response syndrome (SIRS), which may arise in patients with several conditions, including sepsis, burns, major surgery, and severe trauma, with or without infection (Balk, 2014; Chakraborty and Burns, 2022). Dysregulated cytokines and pro-inflammatory mediators may lead to decreased vascular resistance, increased cardiac output, and increased capillary permeability. As a result, these critically ill patients have increased renal vascular flow, leading to better clearance of hydrophilic medications when used with intensive fluid therapy and inotropic drugs (Fuster-Lluch et al., 2008; Sime et al., 2015).

Another possible mechanism is renal function reserve (RFR), which represents the kidney’s ability to increase glomerular filtration rate (GFR) under certain physiological and pathological conditions, including hyperfiltration states, cardiorenal patient conditions, and so on (Hoffmann et al., 2018). The renal stress test is executed to estimate the renal filtration rate based on the baseline and stress glomerular filtration rates, which cause the release of prostaglandins and relaxation factors that increase RFR (Sharma et al., 2014; Kindgen-Milles et al., 2020). RFR and ARC have overlapping populations and mechanisms (Baptista et al., 2020). In addition, some studies have shown that integrating SIRS and RFR may be a comprehensive mechanism in ARC (Bilbao-Meseguer et al., 2018).

Recently, it has been suggested that the concept proposed by Nongnuch is closely related to ARC, defined as “brain-kidney crosstalk” (Nongnuch et al., 2014). Both brain and kidney function can be preserved through autoregulation, according to Dias et al. They found that both PRx (cranial perfusion pressure, intracranial pressure) and CPP (cerebral perfusion pressure) can also be preserved (Dias et al., 2015). According to Udy et al., ARC is observed in traumatic brain-injured patients with normal plasma creatinine levels and may be caused by a hyperdynamic vascular state and increased intracranial pressure (Udy et al., 2015).

## 4 Antibacterial therapy in ARC patients

Accumulating studies have explored the impact of ARC status on antimicrobial exposure and antibiotic dosage recommendations. According to recent research, we provided dosage recommendations for 25 antibiotics (Supplementary Table S1).

### 4.1 Penicillins

#### 4.1.1 Piperacillin-tazobactam

Piperacillin-tazobactam (PIP-TAZ) is an 8:1 β-lactam/β-lactamase inhibitor commonly used as a hydrophilic antibiotic against Gram-positive and Gram-negative aerobic and anaerobic bacteria in critically ill patients presenting signs of infection (Landersdorfer et al., 2012). The kidney eliminates ∼50–60% of piperacillin-tazobactam without much metabolic involvement (Bryson and Brogden, 1994; Hayashi et al., 2010). Like other β-lactams, PIP-TAZ has a time-dependent antibacterial activity, defined by the duration (T) during which the unbound drug concentration must be above the minimum inhibitory concentration (MIC). Clinical efficacy requires a f T > MIC target of 50% for piperacillin (Guilhaumou et al., 2019; El-Haffaf et al., 2021).

However, the therapeutic concentrations are clearly inadequate in ARC patients treated with normal dosing regimens, resulting in poor clinical outcomes. Earlier studies showed that a higher ARC rate led to higher free piperacillin clearance, with failure to achieve 100% f T > MIC(Akers et al., 2014; Udy et al., 2015; Carrie et al., 2018b). Studies about PIP-TAZ dosing adjustments generally did not measure TAZ levels because it was assumed that β-lactamases would remain inhibited throughout the dosing intervals without regard to the exponential decay of the TAZ concentration. Even though it is no longer detectable in the concentrations, TAZ is believed to prolong the susceptibility to piperacillin-induced bactericidal effects even after exposure has ended (Strayer et al., 1994; Lodise et al., 2004). Therefore, recommendations for PIP/TAZ dosing regimens are solely based on PIP concentrations.

Optimizing PIP-TAZ therapy is becoming attractive in populations such as the critically ill. Enhancing exposure times via prolonged or continuous infusion administration for time-dependent antibiotics could present a satisfactory strategy to attain the therapeutic target. For patients at increased risk for augmented renal function, dose increments or continuous infusion may be necessary to increase the probability of achieving therapeutic plasma concentrations (Obrink-Hansen et al., 2015; Beranger et al., 2019). Enhanced dosage of PIP-TAZ could also be considered effective to some extent. For example, higher than licensed doses of PIP-TAZ attained the therapeutic target in patients with CrCl ≥150 mL/min (Besnard et al., 2019). Data obtained with a dynamic hollow-fiber *in vitro* infection model showed that dose increments improved bacterial killing and minimized the development of resistance in patients with normal renal function (Bergen et al., 2016). Extended infusion delivery and continuous infusion regimens were considered sufficient owing to cost savings of ∼50.0–66.7% (Akers et al., 2014). Continuous infusion is most effective in cases of elevated MIC for a bacterial pathogen. However, prolonged infusion requires considering some practical aspects, such as the loading dose and drug stability (Lodise et al., 2007). PIP-TAZ is stable at 37°C for at least 24 h, supporting its suitability for continuous infusion (De Waele et al., 2015). However, continuous PIP-TAZ (16 g/2 g/day) infusion is inadequate for ARC patients, especially those with high CrCl ≥170 ml/min. Monte Carlo simulation (MCS) has been proposed frequently as simulated pharmacokinetic profiles to evaluate the probability of target attainment (PTA) of experimental dosage regimens in attaining prespecified pharmacodynamic targets against specific pathogens{Kuti et al., 2003 #4}{Matsu et al., 2019 #5}. It is widely used in studies on antibiotic dosing regimens, and some cases are reviewed below. MCS indicated that higher doses (20 g/2.5 g/24 h) should be necessary without the additional risk of excessive dosing and neurotoxicity (Carrie et al., 2018a). Continual infusions may not be effective in increasing penetration in target tissues with high MICs, since higher blood concentrations are needed (Roberts et al., 2009; Dahyot-Fizelier et al., 2010).

#### 4.1.2 Amoxicillin/clavulanic acid

Amoxicillin/clavulanic acid is a β-lactam/β-lactamase inhibitor oral or intravenous antibiotic against Gram-positive and Gram-negative bacteria commonly used in critically ill patients presenting signs of infection. Amoxicillin/clavulanic acid is commonly used in typical indications such as community-acquired pneumonia, skin, soft tissue, and abdominal infections (Ceelie et al., 2011). Amoxicillin/clavulanic acid is most readily eliminated in urine (∼60–80% for amoxicillin and 30%–50% for clavulanic acid) after oral administration. Amoxicillin is excreted mainly by tubular secretion, and clavulanic acid is primarily eliminated by glomerular filtration (Todd and Benfield, 1990). Like other β-lactams, amoxicillin/clavulanic acid displays time-dependent antibacterial activity. A pharmacokinetic/pharmacodynamic (PK/PD) index of 40% f T > MIC (MIC, 8 mg/L) has been established for amoxicillin, with 100% associated with better outcomes (Haeseker et al., 2014; Roberts et al., 2014).

Limited published studies are available on recommended amoxicillin/clavulanic acid dosing in critically ill patients with a risk of clinical failure due to ARC. Renal function may be a significant covariate for amoxicillin and clavulanic acid clearance. Underdosing of amoxicillin/clavulanic acid could lead to clinical failure. Enhancing exposure times via prolonged or continuous infusion administration could present a strategy to attain the therapeutic target. A minimum dosing regimen of 25 mg/kg amoxicillin every 4 h (as a 1-h infusion) is recommended for infections in this patient group. To maximize tissue penetration and prevent issues with drug stability, longer infusion times are avoided (De Cock et al., 2015). However, in another study, prolongation of infusion time from 30 min to 2 h achieved favorable target attainment (Anne Fournier et al., 2018; Carrie et al., 2019). Another strategy is to maximize the dosing regimen as much as possible. Population pharmacokinetic (PPK) analysis of amoxicillin in critically ill burn patients showed that increased antibiotic doses were required for ARC patients or cases with higher MIC.

### 4.2 Cephalosporins

#### 4.2.1 Ceftolozane/tazobactam

Ceftolozane/tazobactam (C/T), a combination of a potent antipseudomonal cephalosporin (ceftolozane) with a β-lactamase inhibitor (tazobactam), has activity against many Gram-negative pathogens for complicated intra-abdominal infections, complicated urinary tract infections and hospital-acquired pneumonia (HAP) or ventilator-associated pneumonia (VAP) (López Montesinos et al., 2021). It displays time-dependent antibacterial activity. Ceftolozane/tazobactam (C/T) is mainly excreted via the kidney, thus requiring dose adjustments in patients with varying renal function (van Duin and Bonomo, 2016). The probability of target attainment (PTA) for C/T has been benchmarked at 40% free concentration time above the MIC ( f T > MIC) (Craig and Andes, 2013). The thresholds for optimal ceftolozane and tazobactam were defined as f T > MIC ≥39% (MIC, 4 μg/mL) and >20% (MIC, 1 μg/mL), respectively (Lepak et al., 2014; Sader et al., 2014; Melchers et al., 2016).

Although the concentration of C/T is affected by different renal functional parameters, including ARC, PTA of C/T has little been failed to achieve with 60 min infusion. According to PPK analysis, ARC patients do not require dose adjustments of C/T. However, following patient exposure, steady-state volumes of ceftolozane and tazobactam in plasma and pulmonary epithelial lining fluid were significantly decreased. Ceftolozane and tazobactam also displayed high PTA in simulated plasma and epithelial lining fluid (ELF), irrespective of renal function category, including that of patients with severe ARC (Shorr et al., 2021). Another prospective multicenter study reported higher plasma clearance and lower exposure levels of ceftolozane and tazobactam in ARC patients than in healthy subjects (Nicolau et al., 2021).

The dosing regimen adjusts the administration time into prolonged or continuous infusions. Continuous intravenous infusion of C/Z was successfully administered over 24 h in patients with cystic fibrosis and estimated glomerular filtration rates of 215 mL/min (Elizabeth Davis et al., 2019). Extension of the infusion time was sufficient for therapeutic benefit with an MCS (Natesan et al., 2017). However, with another MCS in patients with different renal functions, including ARC, the approved dosing regimens attained target levels for bactericidal activity at MIC of 4 mg/L (Xiao et al., 2017), which could be attributed to different MIC values. The approved dosing regimens with standard intervals are sufficient to obtain PTA at a low MIC value. However, at higher MIC values, prolonged infusion time may improve efficacy.

#### 4.2.2 Cefiderocol

Cefiderocol, a novel parenteral siderophore cephalosporin, exhibits potent efficacy against most Gram-negative bacteria, including carbapenem-resistant strains of *Pseudomonas aeruginosa*, *Acinetobacter baumannii*, and *Enterobacteriaceae* (Ito et al., 2016; Kohira et al., 2016). It is being developed as a therapeutic drug for CR-GNB infections, including bloodstream infections, nosocomial pneumonia, and complicated urinary tract infection. It displays time-dependent antibacterial activity. About 60%–70% of cefiderocol is excreted unchanged via the kidney, supporting the importance of renal function for clearance of this drug. A bacteriostatic effect at 40%–70% f T > MIC and a bactericidal effect at 55%–80% f T > MIC have been reported in terms of efficacy. Enhancing exposure times via administration of more frequent dosage intervals or prolonged infusion could present a strategy to attain the therapeutic target. Cefiderocol dosage intervals should be shortened for ARC patients to ensure adequate exposure against higher MIC. Clearance of cefiderocol in ARC patients is higher than in patients with normal renal function. Based on MCS, a more frequent dose (every 6 h) was beneficial for ARC patients to attain adequate f T > MIC (Kawaguchi et al., 2018). A dose of 2 g q6h over 3 h of infusion would provide sufficient exposure in patients with ≥120 mL/min CrCl, as estimated with MCS (Kawaguchi et al., 2021), suggestive of improvement in PTA with more frequent dosing and prolonged infusion time of cefiderocol.

#### 4.2.3 Cefepime

Cefepime is an extended-spectrum cephalosporin against Gram-positive and Gram-negative pathogens for treating pneumonia, soft tissue, and complicated urinary tract infections (Suttels et al., 2022). It displays time-dependent antibacterial activity. Cefepime is primarily eliminated through the renal pathway (∼80%) as an unchanged drug (Barradell and Bryson, 1994). In general, cefepime is safe and efficacious, with a goal exposure target of 70% f T > MIC.

Prolonged or continuous infusions are the referenced strategy to optimize the dosing regimen. Daily doses of 2 g in MCS for cases with 140 mL/min CrCl resulted in relatively low PTA. However, intermittent infusion under conditions of renal failure may improve clinical outcomes than intravenous infusion (Liu et al., 2020). For febrile neutropenic patients with normal renal function or ARC, cefepime 2 g t.i.d. with 3 h of infusion was effective in several cases (Yamashita et al., 2016). A study on pediatric patients recommended a regimen of 100 mgkg-1d-1 administered as a continuous infusion to attain higher target values (de Cacqueray et al., 2022).

#### 4.2.4 Ceftazidime/avibactam

Ceftazidime/avibactam (CZA-AVI) is a β-lactam/β-lactamase inhibitor antimicrobial agent to combat increasing antimicrobial resistance among Gram-negative pathogens, predominantly used in serious bacterial infections. It displays time-dependent antibacterial activity. Ceftazidime is not metabolized and is mainly eliminated through urine (Richards and Brogden, 1985), and avibactam is removed unchanged by renal excretion (Lagace-Wiens et al., 2014).

Limited studies to date have focused on the effect of ARC on CZA-AVI therapeutic outcomes. MCS showed that CZA-AVI of 2.5 g achieved PTA (50% f T > MIC) for MIC ≤16 mg/L in patients with normal renal function or ARC (131–190 mL/min) (Stein et al., 2019). Key target attainment remained >95% in 150–180 mL/min and 180–395 mL/min subgroups with a CZA-AVI dosing regimen of 2.5 g q8h, indicating a relatively small increase in antibiotic clearance at higher CrCl. Interestingly, higher CZA-AVI exposure (100% joint target attainment) was reported for Japanese patients’ relative to Caucasian and other reference populations (Li et al., 2019). Both studies demonstrated that routine dosage of CZA-AVI achieved sufficient therapeutic targets in ARC patients. Therapeutic drug monitoring (TDM) of CZA-AVI in four carbapenem-resistant *K.pneumoniae*-infected patients with different kidney statuses indicated that in one patient with CrCl of 194.98 ml/min/1.73m2 treated with CZA-AVI dose of 2.5 g q6h, sputum and urine cultures turned negative after 3 days. Administration of CZA-AVI (2.5 g q8h on the first day) in another subject with higher CrCl of 295.49 ml/min/1.73m2 did not improve infection, leading to the conclusion that ARC patients need higher daily doses of CAZ-AVI(Teng et al., 2022), suggesting that enhanced the dosing internal might be a strategy to attain the PTA Since ceftazidime and avibactam is mainly excreted through the kidney, further research is warranted to establish the impact of ARC on the clinical efficacy of CZA-AVI.

#### 4.2.5 Other cephalosporins

Cefathiamidine is commonly used to treat children with ARC with empirical antimicrobial therapy. It is excreted primarily in an unchanged form by the kidney (>90%) within 12 h after intravenous administration (Tze-ying et al., 1979). Consequently, kidney function plays a significant role in cefathiamidine’s pharmacokinetics. A 70% f T > MIC target was considered a conservative pharmacodynamic endpoint for infants. Simulations in infants with ARC revealed that increasing the dosing frequency could avoid possible toxicity and achieve the pharmacodynamic target (Du et al., 2021).

Ceftazidime, a broad-spectrum cephalosporins with a spectrum of activity, is usually given as 2 g i.v. every 8 h. It is primarily eliminated by glomerular filtration, with elimination half-lives of 1.8–2.3 h in patients with normal renal function. Higher than licensed dosing regimens of β-lactams may be effective and safe in reducing the rate of recurrence and therapeutic failure in critically ill ARC patients (Carrie et al., 2019). In patients with late-onset hospital-acquired pneumonia (HAP), MCS indicated that prolonged infusion of ceftazidime could achieve a high probability of target attainment (Kim et al., 2009). However, this study did not include ARC patients (Kim et al., 2009).

Ceftobiprole, the active moiety of ceftobiprole medocaril, is recommended as a 500 mg 2-h intravenous infusion every 8 h in adults with normal renal function (Murthy and Schmitt-Hoffmann, 2008). As an antimicrobial, it is effective against Gram-positive and Gram-negative pathogens (Farrell et al., 2014). Dose adjustments of ceftobiprole are recommended according to renal function. When treating critically ill patients with ARC, ceftobiprole should be instilled over 4 h for optimal drug exposure (Torres et al., 2016).

Ceftriaxone, one of the most commonly used antibiotics for community-acquired infections, has pharmacokinetic properties, including high binding to albumin, mixed biliary and renal elimination, and long elimination half-life. In earlier studies, trough concentrations were lower in ARC patients (Joynt et al., 2001; Schleibinger et al., 2015). Increasing the dosing regimen may therefore be necessary to obtain PTA of 99% in patients with CrCl ≥150 mL/min (Ollivier et al., 2019). Another multicenter PPK study supported the recommendation of 3–4 g per day, administered in divided doses or as a continuous infusion (Heffernan et al., 2022).

Cefuroxime, a second-generation cephalosporin, is a time-dependent antibiotic frequently used to treat critically ill patients (Vree and Hekster, 1990).In patients with normal renal function, 95% of cefuroxime is excreted unchanged in the urine (Foord, 1976). Continuous infusion of higher doses after the loading dose of cefuroxime has been proposed as a better strategy to achieve therapeutic targets in critically ill patients (Carlier et al., 2014). However, this dosage regimen was insufficient for patients with CrCl ≥300 mL/min (Carlier et al., 2014).

Cefazolin is a narrow-spectrum cephalosporin active against methicillin-sensitive *Staphylococcus aureus* (MSSA) infection. The hydrophilic drug is highly bound to human serum albumin, and eliminated by the kidney (Weis et al., 2019). Among critically ill children with normal and augmented renal function infected with methicillin-susceptible *S. aureus* (MSSA), continuous infusion of cefazolin served as the most feasible scheme to reach the PK target of 100% f T > 4MIC(Salvador et al., 2021).

### 4.3 Carbapenems

#### 4.3.1 Meropenem

Wide-spectrum carbapenem antibiotic meropenem is commonly used in ICU therapy to combat life-threatening infections caused by Gram-positive and Gram-negatives (Mattoes et al., 2004). It is a time-dependent bactericidal antibiotic and unbound concentrations of 100% f T > 1-4MIC are recommended for optimal bacterial killing and avoiding antimicrobial resistance (Abdul-Aziz et al., 2020). It is a hydrophilic drug with a low V (0.3 L/kg) and a very low level of protein binding rate (<5%), mainly eliminated by the kidneys and with a short half-life (Mouton et al., 2000). Standard meropenem dosing may not confer adequate plasma exposure in children with increased renal function. Immunocompetent children with ARC could still achieve 40% f T > MIC following pathogen exposure to the antibiotic at a MIC of 4 mg/L but not >8 mg/L. In ARC patients with critical illness or compromised immune systems, adequate plasma exposure could only be expected at MICs ≤0.25 mg/L when targeting 80% f T > MIC, supporting the urgent need for optimal dosing strategies for children in pediatric intensive care units with sepsis and septic shock receiving renally eliminated antibiotics (Avedissian et al., 2020b). PK/PD target achievement of meropenem is poor in critically ill septic patients with preserved or increased renal function (Gijsen et al., 2022), and dose optimization thus remains an essential requirement.

The first dosing regimen strategy is to use prolonged or continuous infusions to take advantage of the nature of time-dependent antibiotics. After 180 min of infusion, PTA ≥90% remained within the range of 100–300 mL/min CrCl with dosing regimens of 1 g every 8 h and 2 g every 8 h. A 2 g q8h dosing regimen with 180 min infusion was established as the empirical therapy in ARC patients (Tamatsukuri et al., 2018). Prolongation of infusion time (usually at least 3 h) is one of the most efficient ways to achieve the therapeutic target (Kim et al., 2018). However, even when the dose was administered as an extended infusion over a 3 h period, up to 37% of ARC patients were potentially at risk for treatment failure, as this minimum PK/PD target without dose uptitration was not achieved in critically ill patients treated with meropenem (Carlier et al., 2013). Among patients with ventilator-associated pneumonia (VAP), prolonged meropenem infusion was confirmed as a more appropriate strategy than dose elevation in those with ARC (Liebchen et al., 2020; Razzazzadeh et al., 2022).

Maximizing the dosing regimen as much as possible is another strategy to attain the PTA. The meropenem regimen was increased to 2 g q8h to improve clinical outcomes. Therapeutic drug monitoring showed the significant utility of increased meropenem doses in preventing failure to receive sufficient antibiotics (Troger et al., 2012; Selig et al., 2022). However, toxicity should be considered when increasing the antibiotic dose. No data is defined in the literature on toxic levels for meropenem since β-lactam antibiotics were considered safe. Limited studies report the neurotoxicity related to plasmatic levels of β-lactams, with C_min_ concentrations of 64.2 mg/L and 44.45 mg/L increased by 50%, the risk of developing neurotoxicity and nephrotoxicity{Imani, 2017 #1}. Therefore, therapeutic drug monitoring-guided meropenem treatment may be necessary to ensure adequate drug exposure, suggesting a C_min_ therapeutic target for empiric therapy of 8–32 mg/L to enhance efficacy and reduce toxicity{Steffens et al., 2021 #2}.

Combination therapy protocols may be utilized as the third optional optimal dosing regimen. A combination of aminoglycosides with β-lactams demonstrated good bactericidal activity and suppression of resistance in critically ill patients with ARC, supporting its therapeutic potential. Meropenem and ciprofloxacin regimens were administered intermittently due to monotherapy’s failure against *P. aeruginosa* in patients with ARC. *In vitro*, the combination regimens were generally effective at suppressing the resistance of isolates susceptible to one or both antibiotics and promoting synergistic killing. In conjunction with ciprofloxacin, higher than approved daily doses of meropenem may benefit cases infected with more resistant clinical isolates (Agyeman et al., 2021). Another study reported that continuous infusion of meropenem combined with a high dose of daily tobramycin effectively suppressed the regrowth and amplification of less resistant isolates (Yadav et al., 2019).

#### 4.3.2 Other carbapenems

A carbapenem antibiotic, doripenem, is effective against many Gram-positive and Gram-negative pathogens. PTA of f T > MIC of 40% is required for efficacy. ARC, particularly at > 150 mL/min, can induce therapeutic failure if dose adjustment is not considered. Increasing CrCl, 4 h infusions are recommended (Roberts and Lipman, 2013). Another earlier study on a Korean population demonstrated that for cases of ARC or pathogenic infections with doripenem MIC >2 g/mL, 4 h infusions are required to achieve PTA of >90% (Lee et al., 2017).

A PK/PD target of 40% f T > MIC was obtained for imipenem. Despite the high incidence of ARC among burn patients, an imipenem dose of 500 mg q6h was adequate for simulated burns treated via renal-dosed continuous veno-venous hemofiltration (CVVH). However, in cases of suspected ARC (150–200 mL/min) and/or exposure to CVVH, imipenem therapy in burn patients is likely to fail, requiring dose adjustments to 1,000 mg q6h (Por et al., 2021).

The utility of relebactam as a fixed-dose combination with imipenem/cilastatin has been documented. An earlier PPK analysis of imipenem/relebactam included ARC patients with >90% PTA(Bhagunde et al., 2019).

### 4.4 Glycopeptides

#### 4.4.1 Vancomycin

The glycopeptide antibiotic vancomycin (VCM) is used in the treatment of Gram-positive bacteria like methicillin-resistant *S. aureus* (MRSA) and Methicillin-resistant coagulase-negative staphylococci (MRCNS) (Liu et al., 2011). Most of the drug is excreted through the renal route, and >80%–90% is eliminated primarily through urine within 24 h (Rubinstein and Keynan, 2014). Therefore, renal function greatly influences vancomycin pharmacokinetics (da Silva Alves et al., 2017). Therapeutic drug monitoring (TDM) is necessary to maintain a trough concentration between 10 and 20 g/mL to ensure safe and effective dosing. The ratio of the area under the concentration-time curve to the minimum inhibitory concentration (AUC_0–24h_/MIC) was used as the goal for vancomycin treatment optimization, and the target was to achieve a target of AUC_0–24h_/MIC ≥400. If a patient has a severe MRSA infection with an assumed AUC/MIC ratio of 400–600 (MIC of 1 mg/L), trough monitoring should not be performed (Rybak et al., 2020). Thus, performing TDM with AUC_0–24h_/MIC guide dosing is important to ensure effective and safe therapy.

Vancomycin clearance by the kidney is accelerated in ARC patients owing to an elevated creatinine clearance rate, resulting in reduced effectiveness (Bakke et al., 2017; Bilbao-Meseguer et al., 2018; Izumisawa et al., 2019; Chu et al., 2020; Zhao et al., 2021; Scully et al., 2022; Yu et al., 2022). ARC risk is significantly increased by febrile neutropenia or neurosurgery, and clearance of VCM is augmented, resulting in subtherapeutic levels (Hirai et al., 2016; Kim et al., 2016).

Higher doses of VCM may be necessary to achieve sufficient therapeutic effects (Mikami et al., 2022). Data from PK/PD modeling and MCS in children with hematological malignancies showed that at MIC of 0.5 or 1 mg/L, the recommended doses to achieve target AUC_0–24h_/MIC ≥400 were 25–40 and 50–75 mg/kg/d, respectively (Lv et al., 2020). Even at the maximum recommended dose of VCM of 161.9 mL/min/1.73m2 provided to a patient with eGFR, therapeutic efficacy could not be achieved, with consequent treatment failure and a fatal outcome, clearly highlighting the need for early dose adjustment of VCM based on urinary CrCl (Pata et al., 2021). The typical VCM dosage was insufficient and higher doses were also required to achieve adequate exposure in critically ill infants with ARC (Huang et al., 2022). However, patients with higher exposure to vancomycin are more likely to experience toxicity, such as acute kidney injury{Kim et al., 2023 #3}. AUC_0–24h_/MIC ≥400 based on TDM was used to ensure therapeutic effectiveness and safety.

A few studies currently recommend a continuous infusion of vancomycin to improve outcomes. In these reports, loading and continuous infusion were conducted to avoid subtherapeutic serum VCM concentrations in ARC patients (Curth et al., 2015; Villanueva et al., 2019).

A therapeutic switch from VCM to linezolid could improve outcomes since linezolid is mainly eliminated through a non-renal pathway. However, elevated linezolid clearance was recently reported in ARC patients (Barrasa et al., 2020).

#### 4.4.2 Teicoplanin

Teicoplanin is a glycopeptide antibiotic that is active against methicillin-resistant *S. aureus* infections. Approximately 2%–3% of a teicoplanin dose administered intravenously is metabolized, and most of its elimination mainly occurs via the kidney. This drug was approved for three loading doses of 12 mg/kg/d q12 h followed by one maintenance dose of 12 mg/kg/d qd. Based on the TDM, 15–30 mg/L is the probability of achieving the target concentration range. AUC/MIC is the pharmacokinetic/pharmacodynamic (PK/PD) index used to establish teicoplanin efficacy, but the specific PK/PD target for teicoplanin remains to be defined (Byrne et al., 2017). In two previous small-scale clinical studies, patients with MRSA infections had AUC of 750–800 mg h/L (MIC of 1 mg/L) (Craig, 2003; Kanazawa et al., 2011). Patients with neutropenia displayed a 25% increase in clearance compared with non-neutropenia cases. Regimen A was recommended for patients with neutropenia and 600 mg loading and 400 mg maintenance doses were used for ARC. Regimen B (600 mg–400 mg on day 3) was proposed for patients without neutropenia simulated in a PPK model study (Sako et al., 2021).

Few reports to date have focused on dose adjustment of teicoplanin. A high dose might be one of the optional regimens. A loading dose of 800 mg q12h three times with a maintenance dose of 800 mg (severe infection) or 600/400 mg (mild infection) was employed for ARC patients (Hu et al., 2022). The optimal dosing regimens for children with different renal functions were stimulated with the PK model and those with normal or augmented renal function recommended three loading doses of 12 mg/kg q12h, followed by a maintenance dose of 10 mg/kg quarter in die (Gao et al., 2020).

### 4.5 Oxazolidinones

Linezolid, the first member of oxazolidinone antibiotics, has activity against various Gram-positive microorganisms, including methicillin-resistant *S. aureus* (Hashemian et al., 2018). Regardless of a patient’s liver or renal function, the recommended dose of linezolid is 600 mg every 12 h. It is an antibiotic with concentration- and time-dependent activity. Moderate linezolid binding to plasma proteins (31%) has been demonstrated, with ∼65% clearance via a non-renal pathway (Dryden, 2011). Plasma concentrations above MIC (%T > MIC) and AUC_24_/MIC ratio are the PK/PD parameters that best predict clinical efficacy. The recommended PTA is reported as AUC_24_/MIC >80 or %T > MIC >85% (Minichmayr et al., 2017; Barrasa et al., 2019). The correlation between linezolid clearance and creatinine clearance is currently a subject of controversy. A few researchers suggest no relationship between renal function and linezolid pharmacokinetics in critically ill patients, while others believe that renal function affects linezolid pharmacokinetics (Barrasa et al., 2019). Thus, the standard dose regimen may not be the most suitable for all patients.

The status of ARC in critically ill patients significantly affects the pharmacokinetics of linezolid. While only 30%–35% of linezolid is normally excreted in the urine, a significant increase in linezolid clearance and a high risk of underexposure in patients is associated with ARC (Cojutti et al., 2018). PTA (AUC_24_/MIC >80 or %T > MIC >85%) has not been reached in ARC patients. Based on MCS, a regimen of 600 mg Q8h with 30 min infusions did not improve the probability of successful treatment, highlighting the necessity to modify the dosing strategy for ARC patients with increased CL from linezolid. Continuous infusion may thus present a useful strategy (Barrasa et al., 2020). For instance, a 24 h continuous infusion (2,400 mg/day) regimen for ARC patients was reported to achieve sufficient efficacy (Wang et al., 2021). Thus, ARC patients may benefit from continuous infusion and dose increase strategies for the delivery of linezolid, and TDM has potential utility in optimizing PTA (Richards and Brink, 2014).

### 4.6 Aminoglycosides

As part of the empirical treatment of sepsis in critically ill pediatric patients, aminoglycosides, commonly available for Gram-negative bacterial infections, are frequently used in conjunction with β-lactams (Weis et al., 2019). Aminoglycosides are concentration-dependent bactericidal agents with a long-term post-antibiotic effect. Therefore, enhancing the C_max_/MIC or AUC/MIC value is expected to increase the PTA (LeBras et al., 2016). A well-known fact is that critically ill patients exhibit altered organ function and volume of distribution, which significantly reduces their response to aminoglycoside therapy. Given the narrow therapeutic window of aminoglycosides and relatively different degrees of renal elimination, optimizing the aminoglycoside dosage in ARC patients is a considerable challenge (Roberts and Lipman, 2009; Yu et al., 2015).

The incidence of ARC is at least 20% among pediatric intensive care unit (PICU) patients receiving aminoglycosides (Avedissian et al., 2020a). A population-based pharmacokinetic model in the pediatric ICU showed a significantly higher clearance rate (CL) and volume of distribution (Vd) and significantly lower AUC_24h_ in ARC than non-ARC patients (Avedissian et al., 2021). An increase in empirical aminoglycoside dosing in ARC patients is a possible strategy. However, the risk of toxicity should be carefully considered in the younger patient population. Patients with ARC and MIC >1 g/mL should consider combination therapy or drugs other than aminoglycosides (Avedissian et al., 2021). PK/PD values are unlikely optimal when these drugs are administered at standard doses (Avedissian et al., 2021).

Cmax/MIC ratio ≥8 for amikacin is a parameter to predict treatment efficacy. The kidney almost wholly eliminates the drug by glomerular filtration (Marsot et al., 2017). Based on Bayesian algorithms, optimal dosage regimens for this drug could minimize the development of bacterial resistance and maximize the efficacy of antimicrobial therapy limited by underdosing or adverse events from overdosing in patients with different renal function levels (Arechiga-Alvarado et al., 2020).

### 4.7 Fluoroquinolones

The implications of ARC in dosing this class of antibacterial are poorly understood. Levofloxacin, a fluoroquinolone antimicrobial, is commonly used to treat Gram-positive and Gram-negative microorganisms’ infections (Anderson and Perry, 2008). Levofloxacin is a lipophilic drug with almost complete renal elimination (Hurst et al., 2002). PTA has been achieved at AUC_24h_ of 50–150 mg h/L for levofloxacin.

Levofloxacin doses higher than the standard values may be needed to achieve therapeutic effects. Patients with morbid obesity often have augmented renal function caused by glomerular hyperfiltration. Among 15 obese patients intravenously administered 750 mg levofloxacin, three with ARC (measured CrCl of 184.3 mL/min) had lower AUC than the remaining 12 patients with normal renal function (Cook et al., 2011). According to data from a study conducted on severely obese subjects (BMI 40 kg/m^2^), higher doses based on weight are not necessarily effective (Pai et al., 2014).

Ciprofloxacin, an antimicrobial drug used for many infections, is mainly excreted by glomerular filtration and tubular secretion (50%–60%) (Campoli-Richards et al., 1988). For critically ill patients with MIC ≥0.5 mg/L and eGFR >100 mL/min, doses up to 600 mg q6h or greater were estimated. Higher doses than the standard licensed dose was necessary to achieve target attainment for patients with ARC or those infected with less susceptible pathogens (Roberts et al., 2019; Gieling et al., 2020).

### 4.8 Colistin

Colistin is administered intravenously as a cationic lipopeptide antibiotic known as colistimethate sodium (CMS) hydrolyzed *in vivo* into its active component, colistin (Orwa et al., 2001). The drug is a last resort for treating critically ill patients suffering from multidrug-resistant (MDR) systemic Gram-negative infections (Li et al., 2006). Colistin is mainly disposed of via non-renal pathways, while CMS is excreted by the kidney (including renal tubular secretion) (Li et al., 2004; Kassamali et al., 2013). The bactericidal action of colistin is concentration-rather than time-dependent (Michalopoulos and Karatza, 2010). For colistin, the area under the plasma concentration-time curve across 24 h at steady state (AUCss, 24 h) of ∼50 mg h/L is required that equates to a target average steady-state plasma concentration (Css,avg) of ∼2 mg/L for total drug (Tsuji et al., 2019).

Limited studies are available regarding the effects of ARC on colistin therapy. A study on colistin use recommended that colistin-induced acute kidney (AKI) is less common in ARC patients (Aitullina et al., 2019). Dalfino et al. (Dalfino et al., 2015) suggested that ARC patients should be administered increased colistin doses, such as 12 MU daily, in cases where CrCl is >130 mL/min. The apparent clearance of colistin decreased with declining kidney function since a higher amount of CMS was converted to colistin with each dose (Li et al., 2006; Ooi et al., 2019). Owing to the influence of creatinine clearance on the PK of formed colistin, it may be necessary to increase the CMS dose in ARC patients and decrease the dose in patients with renal impairment. Furthermore, in cases where the dose is increased, the possibility of nephrotoxicity or other adverse effects should be considered. Therefore, TDM of the plasma colistin concentration may serve as a useful strategy for dose adjustment.

### 4.9 Fosfomycin

Fosfomycin is a broad-spectrum antibiotic that is active against Gram-positive and Gram-negative bacteria. It is used to treat critically ill patients with multidrug-resistant bacteria. Since fosfomycin is hydrophilic with little protein binding, it is almost entirely excreted via glomerular filtration and is subject to a patient’s renal function. The increase in renal clearance rate in critically ill patients with ARC may lead to low blood concentrations and predispose patients to the risk of treatment failure (Parker et al., 2013). To our knowledge, no clinical reports on the effect of ARC on fosfomycin clearance are documented. In two earlier studies, Cmax was reduced by up to 35% compared with healthy patients, suggesting that critically ill patients are likely to have higher Vd of fosfomycin, resulting in lower Cmax (Frossard et al., 2000; Pfausler et al., 2004).

### 4.10 Daptomycin

Daptomycin, the first of the cyclic lipopeptide class of antibiotics, has a wide range of Gram-positive bacteria, especially MRSA and VRE. About 50% of daptomycin was eliminated by renal excretion (Dvorchik et al., 2003). Earlier PPK analysis of daptomycin disclosed a decrease in t1/2 in critically ill patients with CrCl ≥80 mL/min (Gregoire et al., 2019). Other optimized dose regimens were not proposed in this study.

## 5 Limitations and perspectives

There were some limitations in our study. Most of the included studies are observational single centers studies in a specific population or with different definitions and diagnostic techniques of ARC. Furthermore, there are few or no studies on some drugs excreted mainly through the kidney. Hence, larger samples or multicenter prospective studies are needed to explore these related issues to provide more evidence for optimal dosing regimens and conduct a safe and effective therapy. For those patients with confirmed ARC, individualized strategies are needed for all renally cleared medications.

Most of these studies presented the dosing adjustment regimens based on the in-silicon simulations, lacking clinical research support. A unified solution for dose adjustment guidelines was also not reached an agreement. In the future, more accurate population PK/PD modeling or physiologically based pharmacokinetic (PBPK) modeling will need to be developed, which is confirmed by clinical trials. As an approach to accurately determining the target concentration of drugs, TDM technologies show their advantages in drug dose adjustment. More rapid, convenient, and accurate technologies, such as point-of-care testing (Irving and Gecse, 2022), and semi-automated TDM(Jayanti et al., 2022), are being vigorously developed. Finally, another limitation in this review was the English-language restriction. Studies in other languages were not included. More studies are needed to guide the individualized medication of clinical ARC patients.

## 6 Conclusion

In critically ill patients, ARC is a prevalent phenomenon that significantly affects optimal therapeutic exposure of antibiotics predominantly eliminated by renal excretion. Increased elimination of antibiotics in patients with ARC often results in a high risk of subtherapeutic concentrations and clinical failure. Therefore, optimizing dosing regimens for individual patients should be considered to overcome these limitations. Any delay in achieving therapeutic concentrations will lead to increased morbidity and mortality.

Accurate assessment of renal function is essential to identify patients with ARC. Urinary CrCl measurement is more recommended because the GFR estimated by equations leads to the underdiagnosis of ARC. Regular therapeutic drug monitoring may be necessary to optimize treatment efficacy and minimize the risk of treatment failure and/or the emergence of antibiotic resistance. In this review, 25 antibiotic dosage regimens for patients with ARC and various strategies for optimization of outcomes were summarized, including extended infusion time, continuous infusion, increased dosage, and combination regimens. ARC patients, especially critically ill patients, need to make individualized adjustments to antibiotics, including dose, frequency, and method of administration. Further comprehensive research is required to determine ARC staging, expand the range of recommended antibiotics, and establish individualized dosing guidelines for ARC patients.

## Abbreviations

ARC, augmented renal clearance; CrCl, creatinine clearance; ICU, intensive care unit; sCr, serum creatinine; SIRS, systemic inflammatory response syndrome; RFR, renal function reserve; GFR, glomerular filtration rate; MIC, minimum inhibitory concentration; CVVH, continuous veno-venous hemofiltration; TDM, therapeutic drug monitoring; VCM, vancomycin; PICUs, pediatric intensive care units; PIP-TAZ, piperacillin-tazobactam; C/T, ceftolozane/tazobactam; HFIM, hollow-fiber *in vitro* infection model; MSSA, methicillin-sensitive *Staphylococcus aureus*; MRSA, methicillin-resistant *Staphylococcus aureus*; MRCNS, methicillin-resistant coagulase-negative staphylococci; AUC, area under the curve; CMS, colistimethate sodium; MDR, multidrug-resistant; PPK, population pharmacokinetic.
