# Supplementary material for: Individualized antibiotic dosage regimens for patients with augmented renal clearance
Source: Front Pharmacol. 2023 Jul 26;14:1137975. doi: 10.3389/fphar.2023.1137975 (PMC10410082; doi:10.3389/fphar.2023.1137975)
Supplement: Supplementary file 1 [file Table1.docx]

**Supplementary Table S1. Summary of antibiotic studies pertaining to ARC**

| Author, Year | Study Type | Population | N (patients) | Methods | CrCl  (mL/min) | antibiotics | PD target used | Dosing adjustment regimen | Suggestion basis |
| --- | --- | --- | --- | --- | --- | --- | --- | --- | --- |
| Kevin S. Akers, 2014(Akers et al., 2014) | a cross-sectional study | critically ill surgery and trauma patients | 13 | ARC risk score (≧7 higher risk) | / | Piperacillin-tazobactam | $f$T>MIC≧ 50% | extended infusion dosing | Monte Carlo simulation |
| Mieke Carlier, 2013(Carlier et al., 2013) | a prospective, observational | critically ill patients | 60 | MDRD equation or calculated using urinary creatinine | 55-310 | Piperacillin-tazobactam | $f$T>MIC≧ 50% | extended infusion | clinical study |
| Andrew A Udy, 2015(Udy et al., 2015) | a single-center observational study | critically ill patients with sepsis | 48 | calculated using urinary creatinine | <68,  68 to 114, 115 to 170 and >170 | Piperacillin-tazobactam | 100%$f$T>MIC | / | clinical study |
| Phillip J. Bergen, 2016(Bergen et al., 2016) | in silico simulations | / | / | / | 110，250 | Piperacillin-tazobactam | $f$T>MIC≧ 50% | / | hollow-fiber in vitro infection model (HFIM)+In silico simulations |
| Rajbharan Yadav, 2018(Yadav et al., 2018) | in silico simulations | / | / | / | 250 | Piperacillin-tazobactam | / | / | hollow-fiber in vitro infection model (HFIM)+In silico simulations |
| Cédric Carrié, 2018(Carrie et al., 2018a) | a prospective single-center observational study | ICU patients with severe sepsis | 59 | calculated using urinary creatinine | no ARC (40 ≤ CrCL < 130), moderate ARC (130 ≤ CrCL < 200) and severe ARC  (CrCL > 200) | Piperacillin-tazobactam | 100%$f$T>MIC | higher than standard dosing regimen | Monte Carlo simulation |
| Cédric Carrié, 2018(Carrie et al., 2018b) | letter | ICU patients | 180 | calculated using urinary creatinine or CKD-EPI equation | 83–170 | Piperacillin-tazobactam | / | / | / |
| Kristina Öbrink-Hansen, 2015(Obrink-Hansen et al., 2015) | a prospective observational study | critically ill patients with known or suspected septic shock | 15 | / | / | Piperacillin-tazobactam | 50%$f$T>4MIC and 100%$f$T>MIC | dose increment or prolonged infusion | modeling using NONMEM 7.2 |
| Thibaud Besnard, 2019(Besnard et al., 2019) | letter; a retrospective analysis | intensive care settings | 35 | calculated using urinary creatinine | CLCr≥ 150 | Piperacillin-tazobactam | / | higher than licensed doses | / |
| Agathe Be´ranger, 2019(Beranger et al., 2019) | a prospective study | critically ill children | 50 | Schwartz formula | 29–675 | Piperacillin-tazobactam | 50%$f$T>4MIC and 100%$f$T>MIC | prolonged infusion and continuous infusion | Monte Carlo simulations |
| Pieter A J G De Cock, 2015(De Cock et al., 2015) | a prospective open-label pharmacokinetic study | critically ill children | 50 | calculated using urinary creatinine | / | Amoxicillin/clavulanic acid | 40% $f$T>MIC | 25 mg/kg 4hoursly 1 h infusion | Monte Carlo simulations |
| Cédric Carrié, 2019(Carrie et al., 2019) | a single-center retrospective study | hospital or ventilator-acquired pneumonia (HAP-VAP) patients | 22 | calculated using urinary creatinine | 186 (160–223); 174 (147–200) | Amoxicillin/clavulanic acid | 50%$f$T>MIC | dose increment or increase dosage interval | Monte Carlo simulations |
| Anne Fournier, 2018(Anne Fournier, 2018) | a prospective study | ICU burn patients | 21 | CG equation | 128 (65–150) | Amoxicillin | 50%$f$T>MIC | increased dosages of up to 2 g/4 h or prolonging infusion from 30 min to 2 h | Monte Carlo simulation |
| Andrew F Shorr, 2021(Shorr et al., 2021) | a randomized, double‑blind, phase 3 ASPECT‑NP trial study | hospital‑acquired bacterial pneumonia (HABP) or ventilator‑associated bacterial pneumonia (VABP) | 726 | CG equation | 80-150, 150-180, 180-210, >210 | Ceftolozane/tazobactam | 50%$f$T>MIC of 4 μg/mL ceftolozane;35%$f$T>MIC of 1 μg/mL tazobactam | 3 g administered every 8 h is an appropriate dose for critically ill patients with HABP/VABP and ARC. | Monte Carlo simulation |
| Senthil Natesan，2017(Natesan et al., 2017) | population pharmacokinetic model | MDR Pseudomonas aeruginos- infected patients | / | / | MICs 32 mg/L (CrCl 15–50), 16 mg/L (CrCl 51–120) and 8 mg/L (CrCl 121–180) | Ceftolozane/tazobactam | / | extended infusion of 4–5 h or continuous infusions | Monte Carlo simulation |
| David P Nicolau, 2021(Nicolau et al., 2021) | a phase I prospective multicenter open-label PK and safety study | critically ill patients | 11 | calculated using urinary creatinine | CrCl ≥ 180 | Ceftolozane/tazobactam | / | 3 g with 1 h infusion | clinical study |
| Alan J Xiao, 2017(Xiao et al., 2017) | in silico | virtual patient | 1000 stimulated patients | / | ARC (CrCl, >150 to <200), normal renal function (CrCl, >90 to <150) | Ceftolozane/tazobactam | / | / | Monte Carlo simulation |
| Sarah Elizabeth Davis, 2019(Elizabeth Davis et al., 2019) | case report | a cystic fibrosis (CF) patient | 1 | / | 215 | Ceftolozane/tazobactam | / | a continuous infusion regimen of ceftolozane–tazobactam was successfully used in a CF patient with augmented renal clearance | clinical study |
| Nao Kawaguchi, 2018(Kawaguchi et al., 2018) | a phase 3 study | patients with complicated urinary tract infection or acute uncomplicated pyelonephritis | 238 | CG equation | 7-186 | Cefiderocol | / | a more frequent dose (every 6 hours) | Monte Carlo simulation |
| Nao Kawaguchi, 2021(Kawaguchi et al., 2021) | a phase 3 study | patients with pneumonia, bloodstream infection/sepsis, or complicated urinary tract infection | 425 | CG equation | 5-540 | Cefiderocol | / | a more frequent dose (every 6 hours) | Monte Carlo simulation |
| Jiajun Liu, 2020(Liu et al., 2020) | in silico | simulated patients | / | / | 60, 100, and 140 | Cefepime | 70%$f$T>MIC | 30-minute intermittent infusion | Monte Carlo simulation |
| Yoshiko Yamashita, 2016(Yamashita et al., 2016) | a retrospective study | febrile neutropenia (FN) patients | 123 | CG equation | 104.1±28.8 | Cefepime | / | extended infusion | clinical study |
| [Noémie de Cacqueray](https://pubmed.ncbi.nlm.nih.gov/?term=de+Cacqueray+N&cauthor_id=35605841), 2022(de Cacqueray et al., 2022) | a retrospective study | pediatric intermediate and intensive care unit’s patients | 59 | Schwartz formula | 153 (12-857) | Cefepime | 100%$f$T>MIC | 100 mg.kg-1.day-1 as a continuous infusion | Monte Carlo simulation |
| Cédric Carrié, 2019(Carrie et al., 2019) | a single-center, retrospective observational cohort study | critically ill patients | 17 | calculated using urinary creatinine | 186 [160–223] | Ceftazidime | / | / | / |
| Gary E Stein, 2019(Stein et al., 2019) | a prospective, single-center open-label phase 4 single-arm study | critically ill patients | 10 | CG equation | 103(47-190) | Ceftazidime/Avibactam | 50%$f$T>MIC | current dosing regimens 2.5g is effective | Monte Carlo simulation |
| Jianguo Li, 2019(Li et al., 2019) | five Phase III trials | healthy volunteers and patients | 1975 | CG equation | 8-610 | Ceftazidime/Avibactam | 50%$f$T>MIC | current dosing regimens 2.5g is effective | Phase III trials |
| Xin-Qi Teng, 2022(Teng et al., 2022) | case | Carbapenem-resistant *K.pneumoniae*-infected patients | 4 | / | 194.98, 295.49, 39.79, 170.44 | Ceftazidime/Avibactam | / | a more frequent dose (2.5g q6h) | clinical study |
| Bin Du, 2021(Du et al., 2021) | an open-label single-center PK study | infants (age range, 0.35–1.86 years) with ARC | 20 | / | 132-413 | Cefathiamidine | / | the optimal dosing regimens of 50 mg/kg/day q8 h and 75 mg/kg/day q6 h was required to treat bacteria with a MIC 0.5 and 2 mg/L, respectively. | nonlinear mixed effects modelling (NONMEM) |
| Antonio Torres, 2016(Torres et al., 2016) | congress article | patients in the ICU | / | / | / | Ceftobiprole | / | extending the infusion time of ceftobiprole to 4 h | clinical study |
| Julien Ollivier, 2019(Ollivier et al., 2019) | a prospective single-center observational study | critically ill patients | 21 | calculated using urinary creatinine | ClCr (<150, 150 to 200 and >200) | Ceftriaxone | 100%$f$T>MIC | increased dosing regimen (2g twice a day) allowed obtaining a PTA of 99% for a MIC of 2 mg/L | Monte Carlo simulation |
| Aaron J Heffernan, 2022(Heffernan et al., 2022) | a multicenter population pharmacokinetic study | critically ill patients admitted to the ICU | 36 | / | 104 (85–157.88) | Ceftriaxone | Cmin/MIC of >1 | higher dose and continuous infusion | Monte Carlo simulation |
| Mieke Carlier, 2014(Carlier et al., 2014) | a prospective, open-label pharmacokinetic study | critically ill patients | 20 | calculated using urinary creatinine | 10-304 | Cefuroxime | 65%$f$T>MIC | continuous infusion of higher than normal doses after a loading dose | Population pharmacokinetic analysis and dosing simulations |
| E Salvador, 2021(Salvador et al., 2021) | a prospective study | critically ill children infected with methicillin-sensitive Staphylococcus aureus | 39 | / | / | Cefazolin | 100%$f$T>4MIC | continuous infusion | Monte Carlo simulation |
| Mieke Carlier, 2013(Carlier et al., 2013) | a prospective observational pharmacokinetic study | medical and surgical intensive care unit | 60 | calculated using urinary creatinine | 55 to 310 | Meropenem | 50%$f$T>MIC and 100%$f$T>MIC | when extended infusion to over 3-h infusion, up to 37% of the ARC patients did not achieve this minimum PK/PD target without dose up-titration | clinical study |
| Sean N Avedissian, 2020(Avedissian et al., 2020b) | a retrospective study | critically ill pediatric patients with sepsis | 57 | Schwartz formula | 60–200 | Meropenem | 40%$f$T>4MIC | increased 2-fold to 40 mg/kg/dose every 8 hours | Monte Carlo Simulation |
| Matthias Gijsen, 2022(Gijsen et al., 2022) | a single-center prospective observational PK study | patients with severe sepsis or septic shock | 58 | calculated using urinary creatinine | 64-136 | Meropenem | 100%$f$T>MIC and 100%$f$T>4MIC | poor PK/PD target attainment | clinical study |
| Tatsuro Tamatsukuri, 2018(Tamatsukuri et al., 2018) | a retrospective study | patients with sepsis | 17 | CG equation | 185.6 (144-221) | Meropenem | 90%$f$T>MIC | extended infusion | Monte Carlo simulations |
| Yong Kyun Kim, 2018(Kim et al., 2018) | a retrospective PK study | Korean patients with acute infections | 37 | CG equation | ≤50 or >50 | Meropenem | 40%$f$T>4MIC | extended infusion | in silico simulations |
| Uwe Tröger, 2012(Troger et al., 2012) | case report | intensive care unit patients | 2 | CKD-EPI equation or CG equation | 276, 185 | Meropenem | / | increased dosing regimen and a more frequent dose | clinical study |
| U Liebchen, 2020(Liebchen et al., 2020) | case report | infections with multidrug resistant Acinetobacter baumannii in lung transplant patients | 1 | calculated using urinary creatinine | 156 | Meropenem | / | continuous infusion | clinical study |
| Sareh Razzazzadeh, 2022(Razzazzadeh et al., 2022) | a randomized clinical trial | patients with VAP | 45 | calculated using urinary creatinine | 129.34-188.68 | Meropenem | 50%$f$T>MIC | prolonged meropenem infusion | clinical trial |
| Daniel J Selig, 2022(Selig et al., 2022) | a single-center retrospective study | simulated patients | / | / | 150–250 | Meropenem | 40%$f$T>MIC or 99%$f$T>MIC | a loading dose of 1000–2000 mg infused over 30 minutes to 1hour followed by continuous infusion (3000–6000 mg over 24 hours), or intermittent infusion of 2000 mg every 8 hours | Monte Carlo simulations |
| Akosua A Agyeman, 2021(Agyeman et al., 2021) | in vitro model | 72-hour static-concentration time-kill (SCTK) studies. hollow-fiber infection model (HFIM) | / | / | / | Meropenem+ciprofloxacin | / | increased dosing regimen and continuous infusion | Monte Carlo simulations |
| Rajbharan Yadav, 2019(Yadav et al., 2019) | in vitro model | 72-hour static-concentration time-kill (SCTK) studies. hollow-fiber infection model (HFIM | / | / | / | Meropenem+Tobramycin | / | increased dosing regimen and continuous infusion | Monte Carlo simulations |
| Elaine D Por, 2019(Por et al., 2021) | a retrospective PK study | burn patients | 21 | CG equation | 150 – 250 | Imipenem | 40%$f$T>MIC | increased dosing regimen | Monte Carlo simulations |
| Pratik Bhagund, 2019(Bhagunde et al., 2019) | a population PK analysis of data from 10 completed phases I–III clinical study | adult healthy volunteers and patients with bacterial infections | 837 | simulations | 90-250 | Imipenem/relebactam | / | / | PTA simulations |
| Jason A Roberts, 2013(Roberts and Lipman, 2013) | pharmacokinetic analysis of phase III trial multiple centers | critically ill adult patients with nosocomial pneumonia | 31 | CG equation | average value 137 | Doripenem | 40%$f$T>MIC | extended infusion to over 4-h infusion | Monte Carlo simulations |
| Dong-Hwan Lee, 2017(Lee et al., 2017) | a single-center prospective observational PK study | adult patients (ages, 18 years) with acute infections | 37 | CG equation | 130-170 | Doripenem | 40%$f$T>MIC | extended infusion to over 4-h infusion | Monte Carlo simulations |
| Peter Thomas Scully, 2022(Scully et al., 2022) | a single-center retrospective study | pediatric patients treated for suspected sepsis | 73 | Schwartz formula | 123 (79–180) | Vancomycin | trough level >10µg/mL | / | clinical study |
| Chun-Le Lv, 2020(Lv et al., 2020) | a retrospective single-center study | haematologic malignancy with augmented renal clearance children | 53 | CG equation | 133.07-1283.6 | Vancomycin | AUC0-24h/MIC ≥400 | increased dosing | Monte Carlo simulations |
| Tomohiro Izumisawa, 2019(Izumisawa et al., 2019) | a retrospective single-center study | Japanese hematologic malignancy and non-malignancy patients | 51 | CG equation | ≧120 | Vancomycin | / | / | clinical study |
| Yan-Xia Yu, 2022(Yu et al., 2022) | a retrospective single-center study | patients who received VCM | 388 | CKD-EPI equation | 141.2±16.0 | Vancomycin | AUC0-24h/MIC 400-600 | / | VCM population pharmacokinetic model |
| Sixuan Zhao, 2021(Zhao et al., 2021) | a retrospective single-center study | ICU patients and non-ICU patients developed shock or evidence of  multiple organ failure. | 209 | CG equation | ≧120 | Vancomycin | / | dose should increase together with creatinine clearance until 180 mL/min. | Monte Carlo simulations |
| V Bakke, 2017(Bakke et al., 2017) | a prospective observational study | critically ill patients | 83 | calculated using urinary creatinine | / | Vancomycin | / | dose adjustments in accordance to therapeutic drug monitoring were made to less than half. | clinical study |
| Yang Chu, 2020(Chu et al., 2020) | a prospective study | suspected or documented Gram-positive infections patients | 292 | CG equation | <80, 80–130, ≥130 | Vancomycin | / | / | clinical study |
| Rama Kanth Pata, 2021(Pata et al., 2021) | case report | sepsis secondary to methicillin-resistant Staphylococcus aureus (MRSA) bacteremia | 1 | MDRD formula | 161.9 | Vancomycin | / | treatment failure and subsequent mortality | case report |
| Harald-Morten Curth, 2015(Curth et al., 2015) | case report | pancreatic adenocarcinoma blood culture revealed a bacteremia with Staphylococcus  epidermidis and Enterococcus faecium | 1 | MDRD formula | ≥200 | Vancomycin | / | increased dosing | case report |
| Keita Hirai, 2016(Hirai et al., 2016) | observational study | pediatric patients with febrile neutropenia | 109 | modified Schwartz formula | 159.7 (90.4, 129.9, 191.2, 322.9) | Vancomycin | / | febrile neutropenia significantly enhanced the risk of ARC and was associated with increased clearance of VCM | clinical study |
| A Jeong Kim, 2016(Kim et al., 2016) | a retrospective study | neurosurgical and non-neurosurgical patients | 132 | CG equation | 127.7 ± 45.5 | Vancomycin | / | neurosurgical patients have augmented VCM CL compared with non-neurosurgical patients | clinical study |
| Guang-Ming Huang, 2022(Huang et al., 2022) | a retrospective study | critically ill infants | 66 | Schwartz formula | 81.6-547 | Vancomycin | AUC0-24h/MIC 400-700 | higher dosages | Monte Carlo simulations |
| Helena Barrasa, 2020(Barrasa et al., 2020) | a retrospective study | critically ill patients treated with continuous infusion of vancomycin | 348 | calculated using urinary creatinine | >50 | Vancomycin | / | loading and continuous infusion dose | clinical study |
| Ruben D Villanueva, 2019(Villanueva et al., 2019) | a retrospective study | critically ill trauma patients | 197 | CG equation | 159.1 (115.1–212.1) | Vancomycin | / | optimizing alternative dosing regimens, such as continuous infusions, were suggested | clinical study |
| Ken-ichi Sako, 2021(Sako et al., 2021) | population pharmacokinetic models | neutropenic patients with augmented renal clearance for hematological malignancies | 119 | / | 15–276 | Teicoplanin | target concentration range (15–30 mg/L) | / | population pharmacokinetic models |
| Sasa Hu, 2022(Hu et al., 2022) | a single-center retrospective study | patients treated with teicoplanin | 46 | CG equation | 65.64–266.82 | Teicoplanin | Cmin > 10 mg/L | a loading dose of 800 mg q12h three times for ARC patients, with800 mg needed as a maintenance dose during severe infection, and 600 mg or 400 mg for mild infection | clinical study |
| Liuliu Gao, 2020(Gao et al., 2020) | a prospective pharmacokinetic research | children aged 0–10 years, with different renal functions | 136 | modified Schwartz formula | 118.99 (30.09–280) | Teicoplanin | / | those with normal or augmented renal function should be given three loading doses of 12 mg/kg q12h followed by a maintenance doses of 10 mg/kg quarter in die | population pharmacokinetic models |
| Helena Barrasa, 2020(Barrasa et al., 2020) | a prospective open-label study | critically ill patients, Gram-positive microorganism | 17 in the first part; 26 in the second part | / | 131-179 | Linezolid | AUC24/MIC>80 or %T>MIC>85% | continuous infusion | Monte Carlo simulations |
| Pier Giorgio Cojutti, 2018(Cojutti et al., 2018) | a case report | an intracerebral hemorrhage patient with augmented renal clearance | 1 | / | 131.0 and 160.2 | Linezolid | / | intracerebral hemorrhage ARC patients might be at risk of suboptimal exposure at standard dosages. | case report |
| Xipei Wang, 2021(Wang et al., 2021) | a multicenter, prospective, open-label, observational study | critically Ill patients | 160 | CG equation | 7.5–222.4 | Linezolid | / | a 2400 mg/day 24-h continuous infusion was recommended for ARC patients. | clinical study andMCSs |
| Sean N Avedissian, 2020(Avedissian et al., 2020a) | a retrospective, cohort study | paediatric ICU | 118 | CG equation | 169.1 (123.9–221.3) | Tobramycin or Gentamicin | AUC/MIC and Cmax/MIC >90 | ARC patients experienced significantly higher CL and V and significantly lower AUC24h compared with non-ARC. | clinical study and Bayesian estimation |
| Sean N Avedissian, 2021(Avedissian et al., 2021) | a retrospective observational PK study | critically ill pediatric patients | 123 | modified Schwartz or CG equation | 132 (95.7 to 185) | Tobramycin or Gentamicin | Cmax/MIC 8 and AUC/MIC 70 | ARC patients may benefit from increased aminoglycoside dosing empirically | Bayesian estimation |
| Norma A Aréchiga-Alvarado, 2020(Arechiga-Alvarado et al., 2020) | an observational prospective study | Patients with suspected or proven infection | 63 | CG equation | 130.0±40.6, 140.4±70.3 | Amikacin | Cmax/MIC of>8 AUC/MIC of>75 | increased aminoglycoside dosing | PK/PD simulations based on Bayesian algorithms |
| Manjunath P Pai, 2014(Pai et al., 2014) | a retrospective, cohort study | severely morbidly obese patients | 68 | CG equation | average value 129 | Levofloxacin | AUC24 of 50-150 mg·h/L | doses increment | Monte Carlo Simulation |
| Aaron M Cook, 2011(Cook et al., 2011) | a prospective pharmacokinetic analysis | Cohort 1 consisted of hospitalized patients prescribed 750 mg of levofloxacin as part of  their medical care. Cohort 2 was composed of ambulatory volunteers. | 15 | CG equation or calculated using urinary creatinine | 140.7±64.4 | Levofloxacin | / | accelerated clearance was evident in the ambulatory obese individuals. | clinical study |
| Emilie M Gieling, 2020(Gieling et al., 2020) | an observational prospective multicenter pharmacokinetic study | critically ill patients | 39 | CKD-EPI equation | 78 (23-208) | Ciprofloxacin | AUC/MIC > 125 | 600 mg q6h | Monte Carlo Simulation |
| Jason A Roberts, 2019(Roberts et al., 2019) | a single-center observational PK study | patients with septic shock | 48 | calculated using urinary creatinine | 67 (7-204) | Ciprofloxacin | AUC/MIC > 125 | increased doses | Monte Carlo Simulation |
| Aleksandra Aitullina，2019(Aitullina et al., 2019) | a single clinical center retrospective study | adult patients, admitted to the ICU | 97 | CKD-EPI equation | <50; 50-108; >108 | Colistin | / | ARC patients received higher cumulative colistin doses and had a tendency towards lower incidence of colistin AKI than patients with lower GFR on baseline. | clinical study |
| Lidia Dalfino, 2015(Dalfino et al., 2015) | a prospective observational cohort study | critically ill adult patients (≥18 years old) | 70 | CKD-EPI equation | 133 (82–182) | Colistin | / | / | clinical study |
| Mong How Ooi, 2019(Ooi et al., 2019) | an open-label, non-randomized population PK study | patients under the age of 18 years in the pediatric intensive care unit | 5 | / | 179 (44-384) | Colistin | / | the daily dose of CMS may need to be increased in ARC patients and decreased in patients with renal impairment | Monte Carlo parametric expectation maximization  algorithm |
| Nicolas Grégoire, 2019(Gregoire et al., 2019) | a single clinical center retrospective study | critically ill patients | 24 | MDRD formula | 131 (89–244) | Daptomycin | bacteriostatic (AUCu/MIC>40) or bactericidal (AUCu/MIC>80) | / | clinical study |

ARC, augmented renal clearance; CrCl, creatinine clearance; ICU, intensive care unit; VAP, ventilator‑associated pneumonia

CG equation: Cockcroft-Gault equation; CKD-EPI equation: Chronic Kidney Disease Epidemiology Collaboration equation; MDRD equation: Modification of Diet in Renal Disease equation
